# Accidental alcohol ingestion triggering severe disulfiram-like reaction in a child receiving cefoperazone-sulbactam: a rare case report

**DOI:** 10.3389/fped.2026.1839484

**Published:** 2026-06-17

**Authors:** Haini He, Min Peng, Xiaoliang Liu, Jiao Chen, Li Ma

**Affiliations:** 1 Department of Pediatrics, West China Second University Hospital, Sichuan University, Chengdu, Sichuan, China; 2 Department of Pediatrics, West China Second University Hospital-Tianfu·Sichuan Provincial Children's Hospital, Meishan, Sichuan, China; 3 Key Laboratory of Birth Defects and Related Diseases of Women and Children of MOE, Department of Pediatrics, West China Second University Hospital, Sichuan University, Chengdu, Sichuan, China

**Keywords:** anaphylactoid reaction, cefoperazone-sulbactam, cephalosporin-induced disulfiram-like reaction, children, safety of medicine

## Abstract

**Background:**

The disulfiram-like reaction (DLR) is a potentially life-threatening adverse drug reaction triggered by concomitant alcohol exposure during treatment with certain medications, particularly cephalosporins containing a methyltetrazolethiol (MTT) side chain such as cefoperazone-sulbactam. It is well-documented in adults, however pediatric cases are rarely reported.

**Case presentation:**

We report the case of a 4-year-old boy hospitalized with bronchopneumonia who received intravenous cefoperazone-sulbactam for 7 days with good clinical response. On the 8th day of hospitalization, approximately 10 min after accidentally consuming a small amount of an alcoholic beverage (RIO cocktail, 3%–8% vol), and during the subsequent cefoperazone-sulbactam infusion, the child suddenly developed generalized rash, facial flushing, drowsiness, dyspnea, hypotension (70/50 mmHg), tachycardia (180 beats/min), and prolonged capillary refill time (3 s), consistent with shock. Initially suspected as anaphylactic shock, cephalosporin-induced disulfiram-like reaction (CIDLR) was proposed after the parents revealed the accidental alcohol ingestion. The child was successfully resuscitated with immediate drug discontinuation, oxygen supplementation, fluid resuscitation, and dexamethasone administration. All symptoms resolved within hours, and the child was discharged home two days later with no sequelae at two-week follow-up.

**Conclusion:**

Although the clinical manifestations and temporal association with accidental alcohol ingestion in this case are most consistent with a disulfiram-like reaction, definitive diagnosis is limited by the lack of objective alcohol quantification, and a hypersensitivity reaction cannot be completely excluded. Clinicians should remain alert to both disulfiram-like reactions and hypersensitivity reactions in children receiving cefoperazone-sulbactam, and strict education regarding alcohol avoidance should be provided to caregivers to prevent life-threatening adverse events.

## Introduction

In the pediatric critical care, acute shock is a life-threatening condition. Its causes are diverse and complex. Accurately identifying the type and trigger of shock is crucial for successful treatment (1). Shock in children commonly results from causes such as septic, hypovolemic, cardiogenic and obstructive shock (2). However, in clinical practice, some atypical causes of shock that are triggered by specific drug-substance interactions and can progress rapidly, requiring heightened vigilance from pediatricians. Among these, the cephalosporin-induced disulfiram-like reaction (CIDLR) exemplifies a typical and highly concerning medical emergency. The CIDLR, refers to a condition in which cephalosporins containing a methyltetrazolethiol (MTT) group inhibit hepatic aldehyde dehydrogenase (ALDH), thereby impairing alcohol metabolism and causing acetaldehyde poisoning even with minimal alcohol intake (3). The clinical manifestations of the CIDLR exhibit a spectrum of severity. Mild cases typically present with facial flushing, nausea and vomiting. In contrast, severe cases might involve hypotension, chest pain, coma, convulsions, electrocardiographic abnormalities, arrhythmia, and even life-threatening shock (4).

Cefoperazone-sulbactam is a third-generation cephalosporin that exerts bactericidal effect by inhibiting cell wall synthesis. This drug has a broad antibacterial spectrum and good tolerance. However, diarrhea and rash are the most common adverse reactions associated with the drug (5). The MTT group within the molecular structure of cefoperazone-sulbactam inhibits aldehyde dehydrogenase activity. This inhibition prevents the normal metabolism of acetaldehyde, which may trigger a CIDLR (6). However, the strength of evidence for CIDLR is not entirely consistent across all agents, particularly for those lacking the MTT side chain (7). Nevertheless, for MTT-containing cephalosporins such as cefoperazone-sulbactam, the association with CIDLR is well established (8). On one hand, the causal link between MTT side chain-containing cephalosporins (e.g., cefoperazone, cefmetazole) and CIDLR is supported by *in vitro*/*in vivo* studies and retrospective clinical reviews (9–11). On the other hand, several opposing perspectives should be acknowledged, including the weaker ALDH inhibitory effect of cephalosporins compared to disulfiram, the lack of high-quality prospective studies, and the potential misdiagnosis of CIDLR as anaphylaxis (7, 10).

CIDLR had been frequently reported in adults, it remained a rare occurrence in children (12). This might be related to children's general lack of alcohol consumption, insufficient clinical awareness and reporting bias. Currently, the limited reports of pediatric cases have been mostly related to the ethanol content in the drug preparations, such as the CIDLR caused by the combination of cephalosporins with prednisolone containing ethanol as a solvent (13). Cefoperazone and other cephalosporins with MTT side chains are widely used in pediatric infection treatment, while alcoholic foods, beverages (such as alcohol-containing chocolates, certain fermented drinks), or drug solvents might be overlooked in the pediatric setting (14, 15).

Notably, CIDLRs have been reported in children (12, 13), however, life-threatening shock caused by accidental ingestion of an alcoholic beverage during treatment with cefoperazone-sulbactam has not been previously described in pediatric cases, making this a unique example of severe CIDLR. Through thorough history-taking, the clinicians identified the cause of shock. After timely and appropriate treatment, the child made a full recovery.

## Case presentation

A 4-year-old boy was admitted to the hospital with a 1-day history of cough, wheezing, and fever. On the day prior to admission, the child developed paroxysmal spasmodic cough with rattling sputum, accompanied by wheezing, tachypnea, and fever (peak temperature 38.6℃). Physical examination on admission: temperature 37.9℃, respiratory rate 45 beats per minute, heart rate 156 beats per minute, blood pressure 100/62 mmHg, SpO_2_ 98%. Mental response was normal, complexion and lips were rosy, three concave sign was negative, bilateral lung breath sounds were coarse, bilateral breath sounds were symmetrical, middle and fine moist rales could be heard in the right lung, heart sounds were strong, no murmur, regular rhythm, no obvious abnormalities were found in the remaining physical examinations. The parents denied his history of drug or food allergy, trauma, or surgery. No significant past illness involving the cardiovascular or respiratory systems was identified. The laboratory investigations revealed an elevated high-sensitivity C-reactive protein (hs-CRP) level of 2.661 mg/dL, with normal white blood cell (8.98  ×  10^9^/L), neutrophil percentage (70.4%), lymphocyte percentage (19.9%), hemoglobin level(129 g/L), and platelet count (189  ×  10^9^/L). Sputum culture was negative, and hepatic/renal function and electrolyte panels were unremarkable. The chest CT revealed mild inflammatory changes in the right upper lobe, leading to a diagnosis of bronchopneumonia. The child was treated with cefoperazone-sulbactam for anti-infection and respiratory management.

Seven days later, the child's body temperature returned to normal, and the cough significantly improved. On Day 8, approximately 10 min after accidental alcohol ingestion, cefoperazone–sulbactam infusion was initiated, and symptoms developed within 5 min. Prompt interventions led to rapid and complete resolution. The temporal sequence of critical events on Day 8 is summarized in Supplementary Material 1. During the history-taking process, the clinician again repeatedly asked the family about the child's food intake and exposure to objects recently. The parents then informed the clinician that the child had consumed an alcoholic beverage (RIO, cocktail) 10 min before the infusion of cefoperazone-sulbactam. According to parental report, the child accidentally ingested 5–10 mL of an alcoholic cocktail (alcohol content 3%–8% vol). Based on a mean concentration of 5% and a body weight of 16 kg, the estimated ethanol dose was 12.3–24.7 mg/kg. However, the blood alcohol concentration was not measured in this study. Therefore, the direct correlation between blood alcohol concentration and the occurrence of the CIDLR cannot be quantitatively confirmed. Nevertheless, the judgment of the causal relationship between alcohol exposure and CIDLR is mainly based on the clear temporal association between alcohol intake and reaction onset, as well as the consistency of clinical symptoms with typical CIDLR manifestations, combined with relevant clinical experience and literature support.

Finally, the clinician considered the possibility of the CIDLR. Following the resuscitation, the child was successfully rescued, blood pressure 97/65mmHg, heart rate 100 beats per minute, respiratory rate 20 beats per minute, SpO_2_ 98%. The child's rashes resolved, heart sounds were strong and rhythmical, extremities were warm, and capillary refill time was normal. The complete blood count, liver function tests, kidney function tests, electrolytes, blood gas analysis, echocardiography and electrocardiogram were all normal. Two days later, the child had no fever, occasional cough, shortness of breath, cyanosis, vomiting and diarrhea, and was discharged home. Two weeks later, the child was reexamined in the outpatient clinic and showed no abnormalities.

## Discussion

In clinical practice, DLRs induced by cephalosporins are easily misinterpreted as anaphylactic reactions (13). In the present case, the clinical manifestations and temporal association with accidental alcohol exposure are most consistent with a cephalosporin-associated disulfiram-like reaction. However, a definitive diagnosis cannot be established due to the lack of objective blood alcohol concentration measurement and reliance on parental recall for alcohol exposure, which constitutes a major methodological limitation. Furthermore, despite the absence of typical allergic features, a hypersensitivity reaction cannot be completely excluded, particularly given reports of delayed sensitization and severe reactions following re-exposure to cefoperazone-sulbactam. Therefore, the diagnosis remains presumptive rather than conclusive, and the causal interpretation should be made with caution.

Given that CIDLR and anaphylactic shock can be easily confused in clinical practice, clearly distinguishing between the two is critical for appropriate emergency management (Supplementary Material 2). CIDLR results from acetaldehyde accumulation due to inhibition of aldehyde dehydrogenase by cephalosporins with an MTT side chain following alcohol exposure (3). It typically presents with prominent facial flushing, rare airway edema or urticaria, and hypotension responsive to fluid resuscitation and glucocorticoids (16). In contrast, anaphylactic shock is an IgE-mediated hypersensitivity reaction characterized by acute airway obstruction, urticaria, angioedema, and refractory hypotension requiring epinephrine as first-line treatment (17). In this case, the absence of allergic skin lesions and severe airway involvement, the close temporal relationship to accidental alcohol ingestion, and rapid recovery without epinephrine confirmed the diagnosis of CIDLR. Causality was assessed using the Naranjo Scale, with a total score of 5, indicating a probable adverse drug reaction (18). Importantly, hypotension in disulfiram-like reactions arises from acetaldehyde-mediated vasodilation, not from mast cell or basophil mediator release. Epinephrine does not reverse acetaldehyde accumulation and may even exacerbate tachycardia or precipitate arrhythmias. After excluding anaphylaxis, the patient can be managed with drug discontinuation, oxygen supplementation, intravenous fluid resuscitation, and supportive care.

The mechanism of the CIDLR is that disulfiram and its metabolites irreversibly inhibit acetaldehyde dehydrogenase in the cytoplasm and mitochondria, thereby leading to acetaldehyde accumulation, the toxic primary metabolite of ethanol in the body. Following alcohol consumption, acetaldehyde accumulates, thereby causing the reaction (19). Previous studies in adults have shown that certain medications might cause a CIDLR, including cefamandole, cefoperazone, cephalexin, and long-acting hypoglycemic agents (e.g., chloro sulfonylurea and toluene sulfonylurea) (20). Of course, the severity of CIDLR is also related to the amount of ethanol intake. According to previous studies, at a blood ethanol concentration of 5 mg/dL, the mild reaction might occur with disulfiram (13). As the ethanol intake increases, acetaldehyde accumulates to 0.1–0.2 mg/dL, which might cause cardiac and muscular toxicity reactions, and at 3.5 mg/dL, sudden death could occur (21). It should be noted that these values are derived from prior studies and were not from this case.

Nevertheless, pediatric cases are rarely reported (Table 1). Zheng et al. (2024) reported that a 14-year-old child had developed an atypical DLR with impaired consciousness after taking a high dose of cefixime and consuming alcohol (22). Small SM et al. (2018) also reported the first case to involve a pediatric patient, describing a mild but likely DLR manifesting as facial flushing in an 8-year-old male. The reaction occurred upon receiving a ceftriaxone infusion preceded by a dose of prednisolone elixir (5% ethanol by volume) for presumed community-acquired pneumonia thought to be complicated by an asthma exacerbation (13). Alonzo et al. (2019) reported a case of a 14-year-old patient who experienced a possible disulfiram-like interaction while receiving metronidazole and prednisone intensol solution (23).

**Table 1:** Case report of disulfiram-like reaction in children.

Literature	Patient age	Causative drug	Alcohol source	Clinical manifestations	Severity	Clinical outcome
Zheng et al. (2024) (22)	14 years	Cefixime	Unspecified	Drowsiness, loss of consciousness, cold extremities	Moderate	Discharged with stable vital signs
Small SM et al. (2018) (13)	8 years	Ceftriaxone	Alcohol-containing drug preparations	Facial flushing	Mild	Flushing resolved; no further reactions
Alonzo et al. (2019) (23)	14 years	Metronidazole	Alcohol-containing drug preparations	Tachypnea, oxygen desaturation, agitation, tachycardia	Moderate	Improved within 24 h; no recurrence
Present study	4 years	Cefoperazone-sulbactam	Accidental ingestion of alcoholic beverage	Generalized rash, facial flushing, drowsiness, dyspnea, hypotension, tachycardia, shock	Severe	Symptoms resolved rapidly; discharged without sequelae at 2-week follow-up

Unlike the studies mentioned above, in the present study, the child ingested a relatively small amount of alcohol but still developed disulfiram-like symptoms. In this case report, when the child developed generalized rash, facial flushing, drowsiness, and dyspnea, the cefoperazone-sulbactam infusion was immediately discontinued. Emergency management was initiated, including maintaining the child's airway, administering oxygen via nasal cannula (0.5 L/min), positioning the patient appropriately, rapid fluid infusion (20 mL/kg), intravenous administration of dexamethasone (0.5 mg/kg), and continuous monitoring of vital signs. Approximately 20 min after the intervention, the child's blood pressure and respiratory status gradually improved, and all symptoms were completely relieved within 1 h.

Therefore, when using such medicines, medical staff must clearly inform the child's parents or guardians that the child must avoid any alcoholic substances during treatment and explain the potential risks in detail. At the same time, clinical pharmacists should strengthen such safety warnings in the medication guidance, and medical staff need to urge the guardians to strictly supervise the children's diet. In pediatric nursing practice and safety management, medication safety cannot be ignored (24). Alcohol consumption in children is often unintentional and involves low-dose intake, indicating that awareness of the CIDLRs in clinical nursing work is relatively low. Thus, nurses should also enhance their professional learning and fully understand the risk of CIDLRs caused by the concomitant use of ethanol-containing medicines, solvents, food, and beverages with cephalosporins. In addition, before prescribing cephalosporins in clinical practice, it is necessary to inquire about the child's and family's recent alcohol exposure history and to provide education on the CIDLR is necessary to ensure medication safety.

The limitations of our study include the lack of accurate quantification of alcohol intake, absence of ethanol and acetaldehyde measurements, single-case design (limiting generalizability), and potential recall bias from caregiver-reported alcohol exposure details. Despite these limitations, this case provides valuable clinical information on a rare but severe CIDLR in a pediatric patient.

## Conclusion

Cephalosporins containing an MTT side chain (such as cefoperazone) have been associated with disulfiram-like reactions in children. Although the clinical presentation and temporal relationship to accidental alcohol exposure in this case are most consistent with a disulfiram-like reaction, the diagnosis remains uncertain due to the lack of objective blood alcohol concentration measurement and reliance on estimated alcohol exposure from parental recall. In addition, a hypersensitivity reaction cannot be completely excluded. Therefore, the causal link between alcohol ingestion and this severe reaction cannot be definitively confirmed, and the diagnosis should be interpreted with caution. Pediatric clinicians should remain alert to both disulfiram-like reactions and hypersensitivity reactions in children receiving cefoperazone-sulbactam, provide thorough education to parents and guardians regarding strict avoidance of all alcohol-containing products, and establish strengthened warnings and standardized emergency procedures to improve medication safety in children, as even minimal or unquantified exposure may trigger life-threatening adverse events.

## Data Availability

The original contributions presented in the study are included in the article/Supplementary Material, further inquiries can be directed to the corresponding authors.
