# Supplementary material for: Accidental alcohol ingestion triggering severe disulfiram-like reaction in a child receiving cefoperazone-sulbactam: a rare case report
Source: Front Pediatr. 2026 Jun 17;14:1839484. doi: 10.3389/fped.2026.1839484 (PMC13318874; doi:10.3389/fped.2026.1839484)
Supplement: Supplementary file 1 [file Table1.docx]

**Supplementary material 1 Temporal sequence of events on hospital Day 8**

| **Time** | **Event** |
| --- | --- |
| 11:35 | Patient accidentally ingested **5–10 mL** of alcoholic cocktail (RIO, 3%–8% vol) |
| 11:45 | Intravenous infusion of cefoperazone‑sulbactam started |
| 11:50 | Onset of generalized rash, facial flushing, drowsiness, dyspnea |
| 11:51 | Immediate discontinuation of cefoperazone‑sulbactam infusion |
| 11:51-11:55 | Oxygen supplementation, fluid resuscitation (20 mL/kg), intravenous dexamethasone administered |
| 12:10 | Clinical improvement: blood pressure and heart rate normalized; rash and dyspnea resolved |
| 12:30 | Complete resolution of all symptoms; vital signs stable |
