# Supplementary material for: Accidental alcohol ingestion triggering severe disulfiram-like reaction in a child receiving cefoperazone-sulbactam: a rare case report
Source: Front Pediatr. 2026 Jun 17;14:1839484. doi: 10.3389/fped.2026.1839484 (PMC13318874; doi:10.3389/fped.2026.1839484)
Supplement: Supplementary file 2 [file Table2.docx]

**Supplementary material 2 Differential diagnosis between cephalosporin-induced disulfiram-like reaction (CIDLR) and anaphylactic shock**

| **Feature** | **CIDLR** | **Anaphylactic shock** |
| --- | --- | --- |
| Precipitating factor | Alcohol exposure during cephalosporin (MTT side chain) use | Drug allergy, food allergy, allergen contact |
| Onset interval | Minutes to 1 hour after alcohol intake | Minutes after drug administration |
| Core mechanism | Acetaldehyde accumulation due to ALDH inhibition | IgE-mediated type I hypersensitivity |
| Facial flushing | Very common, prominent | Less common, inconsistent |
| Gastrointestinal symptoms | Nausea, vomiting common | May occur but not prominent |
| Cardiovascular presentation | Hypotension, tachycardia; usually responsive to fluid | Severe hypotension, tachycardia; often refractory |
| Airway involvement | Rare | Common: laryngeal edema, dyspnea, wheezing |
| Skin manifestations | Flushing, rash; no urticaria common | Urticaria, angioedema, generalized pruritus |
| Response to treatment | Improved rapidly after drug cessation, fluid, glucocorticoids | First-line: epinephrine; requires repeated administration |
| Recurrence | No relapse after alcohol avoidance | May recur on re-exposure |
